# COVID-19 and Neurological Impairment: Hypothalamic Circuits and Beyond

**DOI:** 10.3390/v13030498

**Published:** 2021-03-17

**Authors:** Bashair M. Mussa, Ankita Srivastava, Anthony J. M. Verberne

**Affiliations:** 1 Basic Medical Science Department, College of Medicine, University of Sharjah, Sharjah 27272, United Arab Emirates; 2 Sharjah Institute for Medical Research and College of Medicine, University of Sharjah, Sharjah 27272, United Arab Emirates; ankita2112@gmail.com; 3 Department of Medicine, Austin Health, University of Melbourne, Heidelberg 3084, Australia; antonius@unimelb.edu.au

**Keywords:** COVID-19, SARS-CoV-2, neurological manifestations, hypothalamic circuits, olfactory bulb, respiratory centers, hypothalamic–pituitary–adrenocortical axis, viral infection

## Abstract

In December 2019, a novel coronavirus known as severe acute respiratory syndrome coronavirus 2 (SARS-CoV-2) emerged in Wuhan, the capital of Hubei, China. The virus infection, coronavirus disease 2019 (COVID-19), represents a global concern, as almost all countries around the world are affected. Clinical reports have confirmed several neurological manifestations in COVID-19 patients such as headaches, vomiting, and nausea, indicating the involvement of the central nervous system (CNS) and peripheral nervous system (PNS). Neuroinvasion of coronaviruses is not a new phenomenon, as it has been demonstrated by previous autopsies of severe acute respiratory syndrome coronavirus (SARS-CoV) patients who experienced similar neurologic symptoms. The hypothalamus is a complex structure that is composed of many nuclei and diverse neuronal cell groups. It is characterized by intricate intrahypothalamic circuits that orchestrate a finely tuned communication within the CNS and with the PNS. Hypothalamic circuits are critical for maintaining homeostatic challenges including immune responses to viral infections. The present article reviews the possible routes and mechanisms of neuroinvasion of SARS-CoV-2, with a specific focus on the role of the hypothalamic circuits in mediating the neurological symptoms noted during COVID-19 infection.

## 1. Introduction

In the third week of December 2019, a novel coronavirus, now known as severe acute respiratory syndrome coronavirus 2 (SARS-CoV-2), emerged in Wuhan, the capital of Hubei province, China [1]. This virus causes a serious respiratory illness including lung failure and pneumonia that has been officially named COVID-19 by the World Health Organization [2]. SARS-CoV-2 shares a highly homologous sequence with severe acute respiratory syndrome coronavirus (SARS-CoV); hence, its clinical symptoms are similar to those reported for SARS-CoV [3]. The pandemic represents a global health concern, as almost all countries around the world are affected, and more than 79 million confirmed cases were reported by the end of December 2020 [2]. The common clinical features of COVID-19 infection are fever, cough, fatigue, sputum production, headache, hemoptysis, diarrhea, dyspnea, and lymphopenia [4,5]. In addition, patients with this infection exhibit symptoms associated with induced systemic and localized immune responses and increased levels of inflammation [6]. More importantly, a considerable number of affected patients, about 34%, develop neurological symptoms, categorized into central nervous system (CNS) and peripheral nervous system (PNS) symptoms [7]. The clinical features that are related to the former include headache, dizziness, disturbance of consciousness, acute cerebrovascular disease, and epilepsy, whereas symptoms such as decreased taste, decreased smell, and appetite were associated with the latter [7].

The alarming continuous increase in the number of patients who exhibit neurological manifestations reflects a significant neurological impairment and negative impact on the CNS. Therefore, it is critical to understand the central mechanisms associated with COVID-19 infection and to identify the regions within the CNS that may be involved in the neurological pathogenesis of COVID-19 [8]. Based on the array of neurological symptoms, we believe that hypothalamic circuits are key players in the development of the symptoms that are related to the CNS and PNS manifestations of COVID-19. The hypothalamic circuits receive a great deal of input from the olfactory system, which is directly exposed to SARS-CoV-2, and communicate with various nuclei within the CNS and beyond to reach out to the PNS via the hypothalamic–pituitary–adrenocortical axis.

Therefore, the present article reviews the hypothalamic circuits and their potential involvement in neurological manifestations that are associated with viral infection, in particular, COVID-19. In addition, we considered the significant commonalities between SARS-CoV-2 and SARS-CoV infection to propose the potential mechanisms that are responsible for these neurological manifestations.

## 2. CNS and Viral Infection: Transportation into the CNS

Viral infection of the CNS represents a unique challenge that needs to be resolved efficiently by the host’s immune system to avoid irretrievable damage to other crucial systems that maintain vital functions such as consciousness and balance [9,10]. Coronavirus (CoV) was initially recognized as a pathogen of the respiratory tract; however, several studies have demonstrated the presence of SARS coronavirus in brain tissue specimens obtained from patients with SARS-CoV who experienced significant CNS symptoms [11]. Neuroinvasion by CoVs has been documented for almost all the β-CoVs, including SARS-CoV, MERS-CoV, and SARS-CoV-2, and led to the development of a similar range of symptoms including headache, disturbed consciousness, hyposmia, and paresthesia [8,12]. Neurological manifestations of the viral infection depend mainly on the region and the cells of the brain that have been affected. Therefore, infections of the motor or sensory systems lead to abnormalities and distinct physical symptoms compared with limbic infections [13]. In addition, it was found that dopaminergic neurons were more susceptible to SARS-CoV-2 infection compared with cortical neurons or microglia [14].

Experimental evidence for neurotropism and CNS neuroinvasion of CoV was demonstrated using a large panel of human brain autopsy samples, and pathological findings of COVID-19 have revealed brain tissue edema and neuronal degeneration [15,16]. Other studies have shown that intranasal inoculation with CoV in transgenic mice that express the SARS-CoV receptor, angiotensin-converting enzyme 2 (ACE2), in the airways and other epithelia, caused infection in the airways and brain [17]. It is well documented that viral infections enter via different pathways into the CNS, and potential routes have been proposed including the blood brain barrier (BBB), cerebrospinal fluid barrier (CSF), and retrograde transport in neuronal axons. Some doubts have been raised about the involvement of the CSF as a major transportation route for SARS-CoV-2, given that clinical studies failed to detect significant levels of viral RNA in CSF [18].

The BBB and blood CSF barrier (BCSFB) are highly complex networks protecting the CNS parenchyma from toxic materials including viruses. However, they are considered to be the major routes for viral entry into the CNS [19]. Some viruses have adapted to overcome this obstacle by infecting vascular endothelial cells and creating a direct passage across these barriers to enter the CNS [20,21,22]. Additionally, some areas of the CNS such as the choroid plexus and the circumventricular organs including the hypothalamus are not completely protected by the BBB and can serve as virus entry points [23,24]. The use of infected hematopoietic cells as “Trojan horses” is another mean by which viruses can enter the CNS via the blood supply [25,26]. Inflammation-induced breakdown of the BBB and BCSFB due to systemic viral infection also allows viruses to slip through the fenestrations into the CNS [27].

As a second major route of CNS entry, viruses can be transported into the CNS using retrograde axonal transport in peripheral sensory nerves or via dendrites of olfactory sensory neurons that are directly exposed to inspired air in the nose [28]. Moreover, viruses that are inhaled, such as SARS-CoV-2, could quickly move past mucosal epithelial barriers and infect oropharyngeal tissues [12]. It is important to note that while viruses mainly utilize the peripheral nerve route to enter the CNS, they may utilize both routes simultaneously [29,30]. Viruses that remain within cells of the meninges or ventricular lining often induce meningitis, whereas those that infect the CNS parenchyma give rise to meningoencephalitis, encephalitis, or myelitis [31].

Beyrouti et al. recently reported six consecutive cases of acute ischemic stroke due to an exaggerated inflammatory immune response in COVID-19 patients, resulting in impairment of CNS function [32]. Another study has reported the development of acute necrotizing encephalitis as a result of COVID-19 infection, with lesions in areas of the brain associated with consciousness and memory function including hypothalamic circuits [33].

## 3. Hypothalamic Circuits and Viral Infection

**Figure 1:**
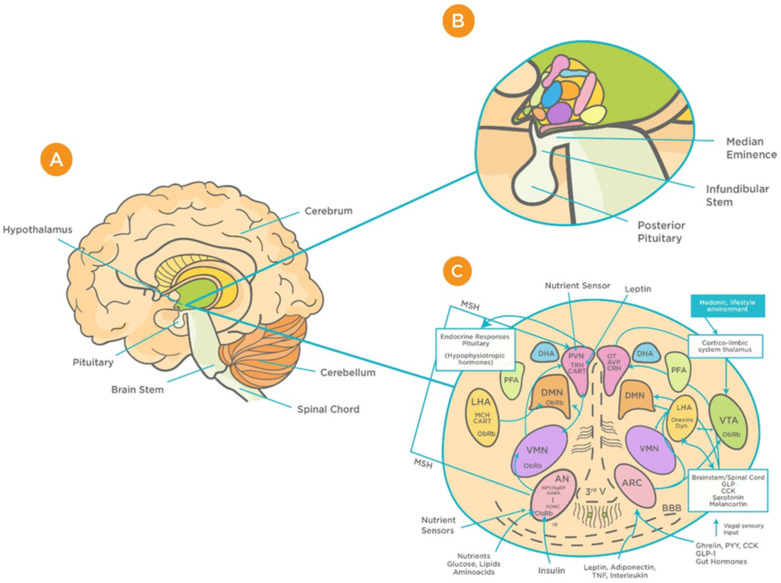
Schematic representation of the functional structure of hypothalamic circuits. (**A**) Location of hypothalamus within the ventral part of the diencephalon. (**B**) Location of hypothalamus in relation to the median eminence, infundibular stem, and pituitary glands. (**C**) Patterns of functional interaction between the hypothalamic nuclei (reproduced with permission from [48]). Abbreviations: AN: arcuate nucleus; AgRP: Agouti-related protein; AVP: arginine vasopressin; BBB: Blood brain barrier; CART: cocaine and amphetamine-regulated transcript; CRH: corticotrophin releasing hormone; GLP-1: Glucagon-like peptide-1; VMN: ventromedial nucleus; OT: oxytocin; DMN: dorsomedial nucleus; PVN: periventricular nucleus; DHA: dorsal hypothalamic area; IR: insulin receptor; PFA: perifornical area; LHA: lateral hypothalamic area; MCH: Melanin-concentrating hormone; MSH: melanocyte-stimulating hormones; CN: suprachiasmatic nucleus; SON: supraoptic nucleus; POA: preoptic area; POMC: Pro-opiomelanocortin; PPY: Polypeptide; ObRb: Leptin receptor; MB: mammillary bodies; ME: median eminence; NPY: Neuropeptide; III-V: third ventricle; TRH, thyrotropin-releasing hormone.

The hypothalamus is a complex structure located at the base of the brain and composed of a large number of cell groups and neuronal circuits that are strongly interconnected [34]. Apart from complex intrahypothalamic connections, the hypothalamus projects to different regions within the brain forming large neuronal networks. The principal hypothalamic nuclei include the paraventricular nucleus (PVN), the perifornical area (PFA), the dorsomedial hypothalamus (DMH), the lateral hypothalamic area (LHA), and the caudal hypothalamus (CH) [35,36,37] (Figure 1). These structures are involved in the regulation of a wide range of physiological functions including respiration, integration of stress responses, thermoregulation, cardiovascular regulation, glycaemia, neuroendocrine regulation, and consciousness [37,38,39,40,41,42,43,44,45,46]. As a relay station, the hypothalamus communicates with almost all regions of the brain, in particular the brainstem, upon receiving vast peripheral sensory inputs from different sources including the olfactory system [47].

Hypothalamic BBB capillaries are fenestrated with fewer tight junctions; thus, they are highly permeable to blood-borne substances, including viruses [49,50]. One such region is the median eminence (ME), which is adjacent to the arcuate nucleus (AN) of the hypothalamus [50]. The unique design of barriers in the hypothalamus “allow the ME and AN to enjoy private milieus,” with the former gaining access to the portal blood, and the latter to the CSF [50]. This makes the BBB at the ME/ARC interface play a major role in determining how hypothalamic neurons are exposed to systemic factors, including viruses [50,51]. Animal studies that have found increased viral load in the brain are suggestive of the virus’s ability to exploit this leakiness and cross the BBB [51]. Such infection can result in deficiencies in essential growth and metabolism hormones. This, in turn, can cause long-term adverse effects on growth, memory, bone health, fertility, pituitary function, and hence quality of life [51,52,53]. Considering these consequences, it is important to address the potential effects that viral infection can have on the functioning of the hypothalamus and its associated neuroendocrine signaling as well as pituitary function—all of which are essential for normal CNS functioning. Given that these findings are observed in other viral infections, for instance the Zika virus [52], it is important to highlight the relevance of these consequences to COVID-19.

Anatomical and electrophysiological studies have shown strong neuronal connections between the olfactory system and the hypothalamus [54]. Anterograde and retrograde axonal tracing studies have revealed that projections from the olfactory system are more prominent to the lateral hypothalamus than to the thalamus [54]. Furthermore, four primary areas in the posterior lateral hypothalamus (anterior olfactory nucleus, olfactory tubercle, piriform cortex, and anterior cortical nucleus of amygdala) were shown to receive this input from the olfactory bulb [54].

Recent studies have suggested different routes for neuroinvasion of CoVs including the peripheral nervous route, hematogenous route, and through the lymphatic system [12]. However, the absence of virus particles in non-neuronal cells in the infected brain casts doubts the involvement of hematogenous or lymphatic routes, especially in the early stages of infection [15,55,56]. Therefore, we believe that the olfactory system may play an important role in the transportation of SARS-CoV-2 into the CNS via the hypothalamus. This hypothesis was supported by recent reports that investigated the neurological manifestations associated with SARS-CoV-2 in patients diagnosed with COVID-19 [57]. It was found that SARS-CoV-2 RNA was detected by RT-PCR in a nasopharyngeal swab specimen but not in CSF, indicating that the neuroinvasion via central routes is most likely via the olfactory system. In addition, imaging studies have shown the involvement of the hypothalamus, mammillary bodies, and dorsal midbrain [57]. This further supported the transneuronal spread of the virus, given the neuronal connection between the hypothalamus and other brain structures.

## 4. Hypothalamus–Olfactory System Crosstalk

It is believed that the olfactory bulb is the main gateway for viral entry into the brain [58]. Previous studies on transgenic mice for the SARS-CoV receptor (ACE2) have investigated the distribution of the viral antigen after intranasal inoculation and confirmed that the site of virus entry into the CNS is the olfactory bulb [58]. Recently, a mouse model expressing human ACE2 (hACE2) by using CRISPR/Cas9 knock-in technology has been developed, and the experiments in this model have demonstrated high viral loads in the brain upon intranasal infection, which further highlights the involvement of the olfactory system in neuroinvasion of SARS-CoV-2 [59]. Simultaneously, viral antigens were detected in other regions of the CNS that possess first- or second-order neural connections with the olfactory bulb. These include the cerebral cortex, basal ganglia, the midbrain, and the hypothalamus [17,58]. The suggested mechanisms for CoVs invasion include internalization in nerve terminals by endocytosis, retrograde transportation, axonal transport, and transsynaptic spread to other brain regions [60].

**Figure 2:**
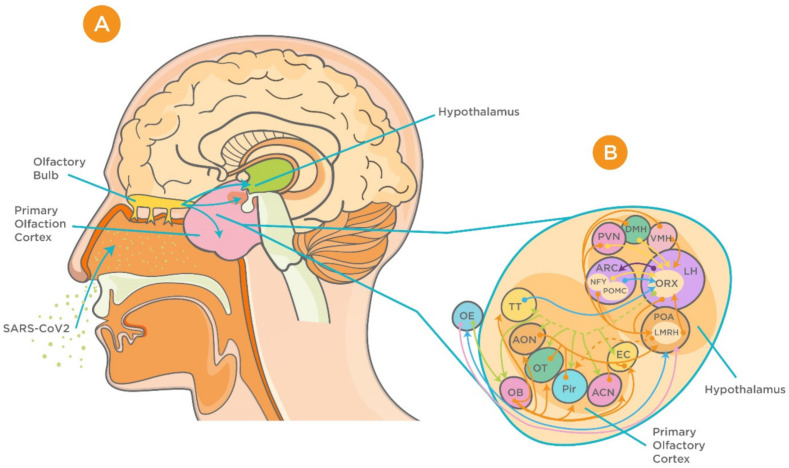
Proposed neuroinvasion of severe acute respiratory syndrome coronavirus 2 (SARS-CoV-2) and interrelationship between the hypothalamus and olfactory system. (**A**) Sagittal view of olfactory bulb, primary olfactory cortex, and the hypothalamus. (**B**) Complex connection between hypothalamic circuits and primary olfactory cortex (reproduced with permission from [70]). Abbreviations: ACN, amygdaloïd cortical nucleus; AON, anterior olfactory nucleus; ARC, arcuate nucleus; DMH, dorsomedial nucleus of the hypothalamus; EBOP, extra bulbar olfactory pathway; EC, entorhinal cortex; LH, lateral hypothalamus; OB, olfactory bulb; OE, olfactory epithelium; OT, olfactory tubercle; Pir, Piriform cortex; POA, preoptic area; PVN, paraventricular nucleus of the hypothalamus; TT, taenia tecta; VMH, ventromedial nucleus of the hypothalamus.

It has been demonstrated that the hypothalamus has complex anatomical and functional connections with the olfactory bulb. Several neuropeptides including gonadotropin-releasing hormone, neuropeptide Y, leptin, adiponectin, and orexins are involved in modulation of these connections [61,62,63,64,65,66] (Figure 2). In addition, studies on influenza viruses, which share several commonalities with the CoVs, have demonstrated high viral antigen expression in the olfactory bulb accompanied by cytokine induction within the bulb and hypothalamus upon intranasal infection [67]. It is noteworthy that these cytokines, which include tumor necrosis factor alpha (TNF-α) and interleukin 1β (IL-1β), play a critical role in triggering acute-phase responses to viral infection [68]. In agreement with previous reports, more recent evidence has demonstrated MRI alteration in the olfactory cortex of patients with COVID-19, indicating the involvement of the olfactory system in the viral neuroinvasion [69]. This became more evident using 3- and 2-dimensional fluid-attenuated inversion recovery images, which showed cortical hyperintensity in the right gyrus rectus and hyperintensity in the olfactory bulbs [69].

## 5. Hypothalamic Nuclei and Modulation of Respiration

The central role of the hypothalamus in the modulation of respiration is well-documented. As early as 1962, lesion and transection experiments in cats demonstrated that inactivation/destruction of the hypothalamus led to a significant decrease in ventilation in awake cats [71]. Subsequent experiments using more sophisticated approaches, such as electrophysiology, neuropharmacology, and brain mapping, have shown that the paraventricular nucleus (PVN), perifornical area (PFA), dorsomedial hypothalamus (DMH), and lateral and posterior hypothalamus are the primary hypothalamic regions that are involved in respiratory modulation [37]. The PVN communicates with several brain regions that are considered critical for driving baseline respiration and control of breathing, including the nucleus ambiguus (NA), the nucleus of the solitary tract (NTS), the ventrolateral medulla (VLM), and the dorsal motor nucleus of the vagus [35,72,73,74]. In addition, the PVN is regarded as a prominent structure in responding to hypoxia via the activation of the chemoreflex [74,75,76]. Other important hypothalamic regions are the PFA and the DMH, which also possess several neural connections with brainstem structures such as the NTS and the VLM and are prominently involved in the regulation of respiratory activities in response to stress in animals and humans [43,77,78]. In addition, it has been shown that both the lateral and posterior hypothalamus are involved in central regulation of coordinated respiratory activities [79]. Great emphasis has been placed on the role of the latter in modulating respiratory responses to stressful stimuli including hypoxia and hypercapnia [80,81]. These are considered as main features of the respiratory distress that is associated with COVID-19 [5]. Although it is well-known that the main cause of such distress is the dysfunction of the pulmonary system and not the CNS, our hypothesis is that the failure of the respiratory centers within the hypothalamus may contribute to the outcomes [82]. Further experimentation is required to confirm these suggestions.

On the other hand, recent epidemiological data regarding COVID-19 has revealed that the median time from the first symptom to dyspnea was around 5 days, and 8 and 10 days to hospital admission and intensive care, respectively [83]. Although this is certainly due to viral incubation time prior to pulmonary complications, we can also suggest that the latency period supports the view that the virus can invade and impair the function of brain regions that are involved in the regulation of respiration [60,79].

## 6. ACE2 Expression in the Hypothalamus

ACE2, a homologue of angiotensin-converting enzyme (ACE), catalyzes the conversion of angiotensin II (AII) to angiotensin-(1–7) peptide and is known to be highly expressed in the hypothalamus [84]. Within the hypothalamus, ACE2 has also been implicated in attenuating hypertension, and the balance between hypothalamic ACE2 and ACE is known to be a determinant of blood pressure [85,86,87]. Furthermore, ACE2 overexpression reduces local inflammation in the hypothalamus, whereas elevated AII levels induce inflammation, subsequently enhancing the mRNA levels of proinflammatory cytokines—TNF-α, IL-1β, and IL-6 [87,88]. Interestingly our recent work showed that hypothalamic miRNAs, small nucleotides that control the regulation of gene expression at the translational level, have binding sites and strong binding capacity against ACE2 and transmembrane serine protease 2 (TMPRSS2) [89].

Both subunits (S1 and S2) of the SARS-CoV are known to bind to the human receptor of ACE2, which is present on non-immune cells (endothelial cells, respiratory and intestinal epithelial cells, kidney cells, and cerebral neurons) as well as on immune cells (alveolar monocytes/macrophages) [90]. Binding of the S2 subunit to the ACE2 receptor leads to its down-regulation, which, in turn, results in excessive AII production coupled with an increase in the permeability of the pulmonary vasculature [90,91]. Amplification of the proinflammatory system due to binding of SARS-CoV-2 to ACE2 has been shown to predispose elderly persons and those with cardiovascular disease to a greater risk of COVID-19 infection and associated severity and mortality [92,93]. A similar profile is also observed in people suffering from hypertension and diabetes. These patients are usually given the ACE inhibitor/AT1R blocker (ACEI/ARB) treatment to increase ACE2 levels. This, however, is exploited by the SARS-CoV-2 binding to ACE2, causing further reduction in ACE2 cell surface expression, upregulating AII signaling in the lungs, and yielding acute lung injury [93]. Such hyperactivity of the ACE–AII–AT1R axis in the hypothalamus, where the ACE2 protein levels are normally low, makes it more susceptible to dysfunction. Expression of ACE2 in sustentacular cells of the nose that transfer odor from the air to sensory neurons has also been implicated in causing partial or total loss of smell, which is one of the first symptoms of COVID-19 infection [94]. This is further enhanced because of the advantage of having a leaky BBB around the hypothalamus for transportation of neuroendocrine signaling molecules within the body.

## 7. Hypothalamic–Pituitary–Adrenocortical Stress Response to Viruses

The involvement of the hypothalamus in the regulation of several hemostatic functions reaches out beyond the central environment. One of the main responses of the hypothalamus to various homeostatic challenges is the stress response, which is considered an integrated reaction to stressors such as viral infections [38]. The stress response is primarily triggered by activation of hypothalamic neurons within the PVN and release of corticotrophin releasing hormone (CRH). CRH stimulates corticotrophs, the corticotropin-producing cells within the anterior pituitary gland, to synthesize and secrete adrenocorticotropic hormone (ACTH), which, in turn, stimulates the adrenal gland to release cortisol [95].

**Figure 3:**
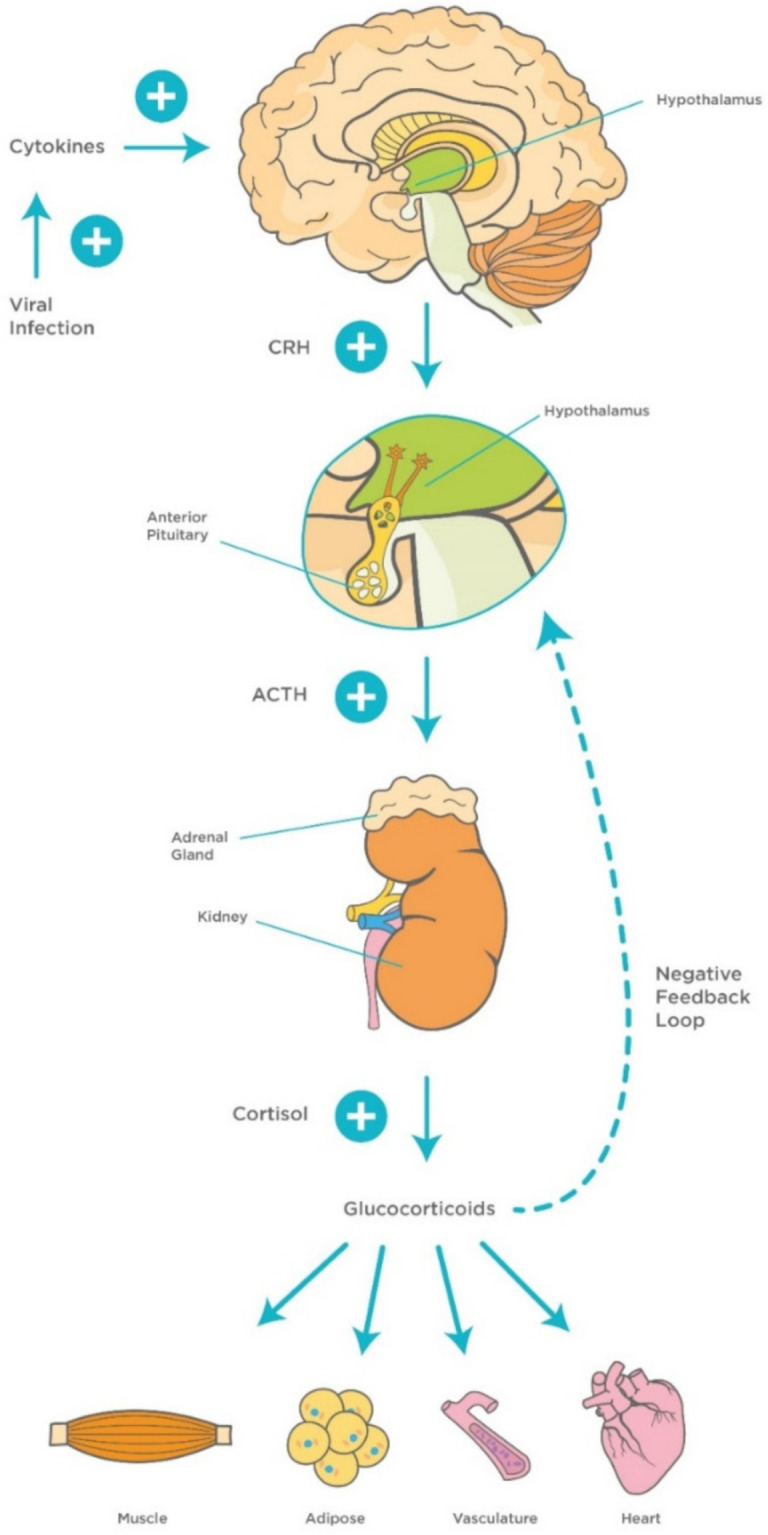
Mechanisms of hypothalamic–pituitary–adrenocortical (HPA) axis during viral infection. Viral infection activates innate proinflammatory cytokines (TNF-α, IL-1, and IL-6) and interferons, and late acquired T cell cytokines (IL-2 and IFN-γ), which activate the HPA axis and release of GCs. This, in turn, stimulates a negative feedback loop to control the immune response. Abbreviations: ACTH, adrenocorticotropic hormone; CRH, corticotropin-releasing factor, TNF-α, Tumor Necrosis Factor-alpha; IL-1, Interleukin-1; IL-6, Interleukin-6; IL-2, Interleukin-2; IFN- γ, Interferon-gamma; GCs, glucocorticoids.

It is well-documented that the hypothalamic–pituitary–adrenocortical (HPA) axis plays a vital role in modulating the host’s susceptibility to viral infections. It has been found that high levels of proinflammatory cytokines, particularly IL-1β, IL-6, and TNF-α activate the HPA axis during the early stages of viral infections, which, in turn, stimulate the release of adrenal glucocorticoids (GCs) to suppress aggressive inflammatory attacks and regulate the immune response [96,97]. Other studies have highlighted the role of different proinflammatory cytokines such as IL-1 in the stimulation of a virus-induced stress response [98]. This represents the rationale for using GCs such as dexamethasone as a potential therapeutic agent for virus-induced respiratory diseases including COVID-19 [99,100] (Figure 3). In addition, GCs are involved in a more vital role, namely the modulation of downstream acquired immune responses, by causing a shift in immune responses from cellular (Th1/inflammatory) to humoral (Th2/anti-inflammatory) type [101]. Furthermore, GCs protect the homeostatic systems against an uncontrolled immune response by stimulating a negative feedback loop that acts directly on the pituitary gland.

It is noteworthy that SARS-CoV was identified in the adrenal glands, suggesting a direct cytopathic effect of the virus [102]. In addition, autopsies of SARS-CoV patients have shown degeneration and necrosis of the adrenal cortical cells; therefore, it was suggested that both SARS-CoV and SARS-CoV-2 may manipulate the stress response and, subsequently, cortisol dynamics [94]. This is considered as one of the main immunoinvasive strategies used by the virus to suppress the host’s response [3,102]. More studies with SARS patients have provided evidence for central hypocortisolism and low dehydroepiandrosterone sulfate levels, indicating damage of the hypothalamic–pituitary circuits [103,104]. On the other hand, lymphopenia is considered as a key hematological feature of COVID-19 and is strongly associated with HPA activation and GCs levels [104,105]. The latter leads to a cascade of events including induction of splenic atrophy, T cell apoptosis, and deficiency of natural killer cells which, in turn, shift hematopoietic stem cell proliferation toward the myeloid lineage resulting in lymphopenia [106,107].

## 8. Future Directions

The present review highlights the importance of the hypothalamic networks in the viral infection processes and suggests a potential role for their involvement in the development of CNS symptoms that are associated with COVID-19. Our previous work has shown that hypothalamic microRNAs play a key role in multiple functions, including food intake, energy balance, and glucose homeostasis [39]. Therefore, we believe that future studies should focus on the investigation of the role of these microRNAs in the pathogenesis of COVID-19. Recently, we have conducted in silico analyses that revealed potential hypothalamic microRNAs that can be used to identify potential therapeutic targets to treat neurological symptoms in COVID-19 patients via the regulation of ACE2 and TMPRSS2 [89]. The presence and expression of the latter may explain the selectivity of having CNS symptoms in some patients and not in others. Although the knowledge about the predisposing factors of neurological dysfunction associated with COVID-19 is limited, we can speculate that the following factors may play a role: preexisting medical conditions, medications, amount of viral exposure, and sub-strain of virus [108].

## 9. Conclusions

Neurological impairment that is associated with COVID-19 is most likely related to SARS-CoV-2 neuroinvasion. It is crucial to highlight that hypothalamic circuits represent an entry point for the virus via the olfactory bulb and communicate with vital respiratory networks. The hypothalamus also reaches out beyond the CNS to the periphery via the hypothalamic–pituitary–adrenocortical axis. Reviewing the aspects of the effects of COVID-19 on the CNS draws attention to the importance of establishing a focused research effort to identify the exact hypothalamic circuits that may be involved in the development of neurological impairment associated with COVID-19 infection.

## Abbreviations

ACE2	Angiotensin-converting enzyme 2
ACTH	Adrenocorticotropic Hormone
AII	Angiotensin II
AgRP	Agouti-related protein
AT1R	Angiotensin type 1a receptor
AVP	Arginine vasopressin
BBB	Blood brain barrier
BCSFB	Blood cerebrospinal fluid barrier
CART	Cocaine and amphetamine-regulated transcript
CCK	Cholecystokinin
CH	Caudal hypothalamus
CNS	Central nervous system
COVID-19	Coronavirus disease 2019
CRH	Corticotrophin releasing hormone
DNH	Dorsomedial hypothalamus
GCs	Glucocorticoids
GLP-1	Glucagon-like peptide-1
IL-1β	Interleukin 1β
IR	Insulin receptor
LHA	Lateral hypothalamic area
ME	Median eminence
MCH	Melanin-concentrating hormone
MSH	Melanocyte-stimulating hormones
NA	Nucleus ambiguus
NTS	Nucleus of the solitary tract
NPY	Neuropeptide
ObRb	Leptin receptor
OT	Oxytocin
PFA	Perifornical area
PNS	Peripheral nervous system
POMC	Pro-opiomelanocortin
PVN	Paraventricular nucleus
PYY	Polypeptide
SARS-CoV-2	Severe acute respiratory syndrome coronavirus 2
TNFα	Tumor necrosis factor alpha
TRH	Thyrotropin-releasing hormone
VLM	Ventrolateral medulla
